# Cessation of methotrexate and a small intestinal resection provide a good clinical course for a patient with a jejunum perforation induced by a methotrexate-associated lymphoproliferative disorder: a case report

**DOI:** 10.1186/s12957-020-02114-0

**Published:** 2021-01-02

**Authors:** Masahiro Nomura, Ryusuke Sumiya, Hayato Ono, Takeshi Nagai, Keigo Kumazawa, Atsushi Shimizu, Daisuke Endo, Nobuyoshi Aoyanagi

**Affiliations:** Department of Surgery, Kohnodai Hospital, National Center for Global Health and Medicine, 1-7-1 Konodai, Ichikawa-shi, Chiba 272-8156 Japan

**Keywords:** Methotrexate, Lymphoproliferative disorder, Intestinal perforation, Jejunum, Epstein–Barr virus, Case report, Epstein–Barr virus-positive mucocutaneous ulcer, EBVMCU

## Abstract

**Background:**

Methotrexate (MTX) is a frequently used drug in the treatment of rheumatoid arthritis (RA), but occurrences of lymphoproliferative disorders (LPD) have been reported in patients undergoing an MTX regimen. Almost half of the patients with methotrexate-associated lymphoproliferative disorders (MTX-LPD) have extranodal lesions; moreover, although extremely rare, digestive tract perforations resulting from the extranodal lesions of MTX-LPD have also been reported.

**Case presentation:**

We describe the case of an 81-year-old woman with RA who had been prescribed MTX at 6 mg per week for the past 11 years. She was admitted to our hospital with occasional abdominal pain and was first diagnosed with enteritis. Her abdominal pain did not improve, and a computed tomography scan showed abdominal effusion and free air in the abdominal cavity. She was diagnosed with a digestive tract perforation and underwent emergency surgery. The perforation site was identified in the jejunum, and she underwent small intestinal resection around the perforated region. The pathological findings showed an ulcer in the jejunum and infiltration of large atypical lymphocytes around the perforated region. An immunohistochemical examination revealed the expression of a cluster of differentiation 20 and latent membrane protein 1. Considering the patient’s history of RA treated with MTX, she was diagnosed as having Epstein–Barr virus (EBV)-related MTX-LPD with a histological diagnosis of EBVMCU. MTX was discontinued after the surgery, and her soluble interleukin-2 receptor (sIL-2R) levels had returned to normal 1 year later. She has had a good course for the 2 years since surgery and remains asymptomatic with no recurrence of MTX-LPD, as confirmed by the sIL-2R levels.

**Conclusion:**

We experienced a rare case of the jejunum perforation induced by MTX-LPD. Since only a few cases have been reported of a patient with small intestinal perforation induced by MTX-LPD, further research is necessary to evaluate the clinicopathological features of MTX-LPD. The patient had disease remission after surgery and by discontinuing MTX treatment; our case did not require chemotherapy. EBV-positive patients, especially those with a pathological presentation of EBVMCU, could have a higher likelihood of remission, which could have been a factor in the present case.

## Background

Methotrexate (MTX) is a key drug in the treatment of rheumatoid arthritis (RA), and one of the adverse effects can be lymphoproliferative disorders (LPD). However, the incidence of methotrexate-associated lymphoproliferative disorders (MTX-LPD) is extremely rare, reported as 0.00168/person-year [1]. MTX-LPD was categorized with other iatrogenic immunodeficiency-associated lymphoproliferative disorders (Oii-LPD) in the revised 2017 fourth edition of the World Health Organization’s (WHO) *Classification of Tumours of Haematopoietic and Lymphoid Tissues* [2]. Almost half of the patients with MTX-LPD had extranodal lesions associated with the brain, lungs, kidneys, liver, and bone marrow [3]. Extranodal lesions in the digestive tract are rare, but a few cases of patients with small intestinal perforations resulting from extranodal lesions of MTX-LPD are reported [4]. Although chemotherapy should be administered to patients who have progressive LPD, spontaneous LPD regression after MTX cessation without chemotherapy is often achieved in up to half the cases of MTX-LPD after ending MTX treatment [5]. Here, we describe a rare case of a patient with a jejunum perforation induced by MTX-LPD who underwent a partial small-intestine resection and had a remission after cessation of MTX.

## Case presentation

An 81-year-old woman was admitted to our hospital due to occasional abdominal pain. She had a medical history of RA for the previous 14 years, hypertension, and nonalcoholic fatty liver disease. To control her RA, she had been taking MTX (6 mg per week) and prednisolone (5 mg/2.5 mg every other day) for the past 11 years. A physical examination revealed pain throughout her abdomen with rebound tenderness on the left side; however, she had no fever, and her abdomen was soft and flat.

Laboratory findings included a white blood cell count, 4400/μL (reference range, 3300–8600/μL); lymphocyte count, 572/μL (13%) (reference range, 18–50%); hemoglobin, 12.8 g/dL (reference range, 11.6–14.8 g/dL); platelet count, 240,000/μL (reference range, 158,000–348,000/μL); albumin, 3.6 g/dL (reference range, 4.1–5.1 g/dL); lactate dehydrogenase, 296 U/L (reference range, 124–222 U/L); and C-reactive protein, 1.91 mg/dL (reference range, 0.00–0.14 mg/dL). An abdominal computed tomography (CT) scan showed small-intestine edema, and her initial diagnosis was enteritis.

**Fig. 1:**
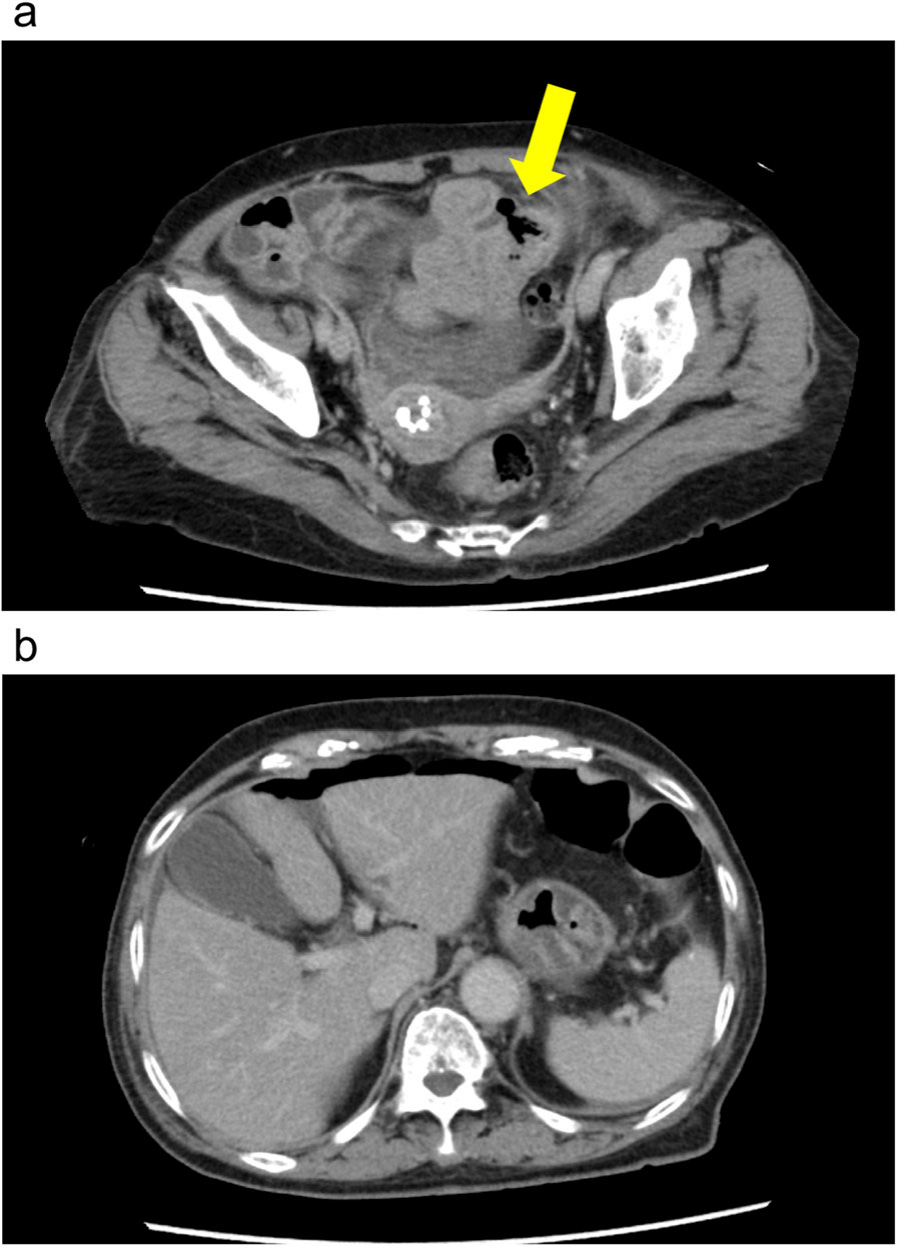
Representative images of CT scan on hospital day 3. **a** Perforation site (arrow) of the small intestine and abdominal effusion. **b** Free air in the abdomen. CT computed tomography

**Fig. 2:**
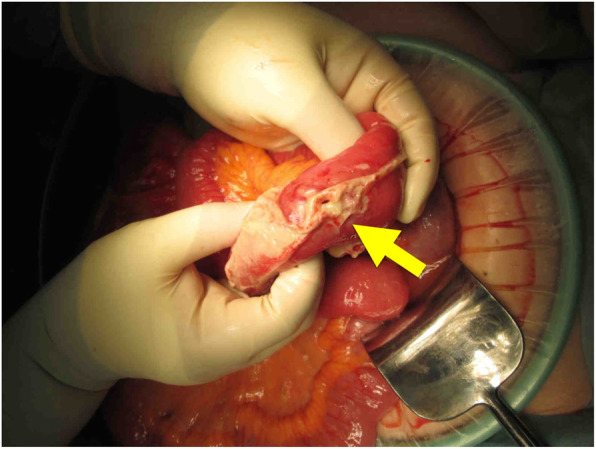
Intraoperative findings revealed that the perforation site (arrow) was in the jejunum, about 30 cm anal-side to the Treitz ligament

Antibiotic therapy was administered on the second day of her hospital stay because her white blood cell count increased from 4400 to 23,000/μL and her temperature increased from 35.9 to 38.5 °C. The patient’s abdominal pain also failed to improve. On the third day, the CT scan showed abdominal effusion and free air in the abdominal cavity (Fig. 1); she was diagnosed with a digestive tract perforation and peritonitis, and emergency surgery was performed. Intraoperatively, the perforation site was identified in the jejunum (about 30 cm anal-side to the Treitz ligament); the patient underwent a partial resection of the small intestine, and intraperitoneal irrigation was performed (Fig. 2).

**Fig. 3:**
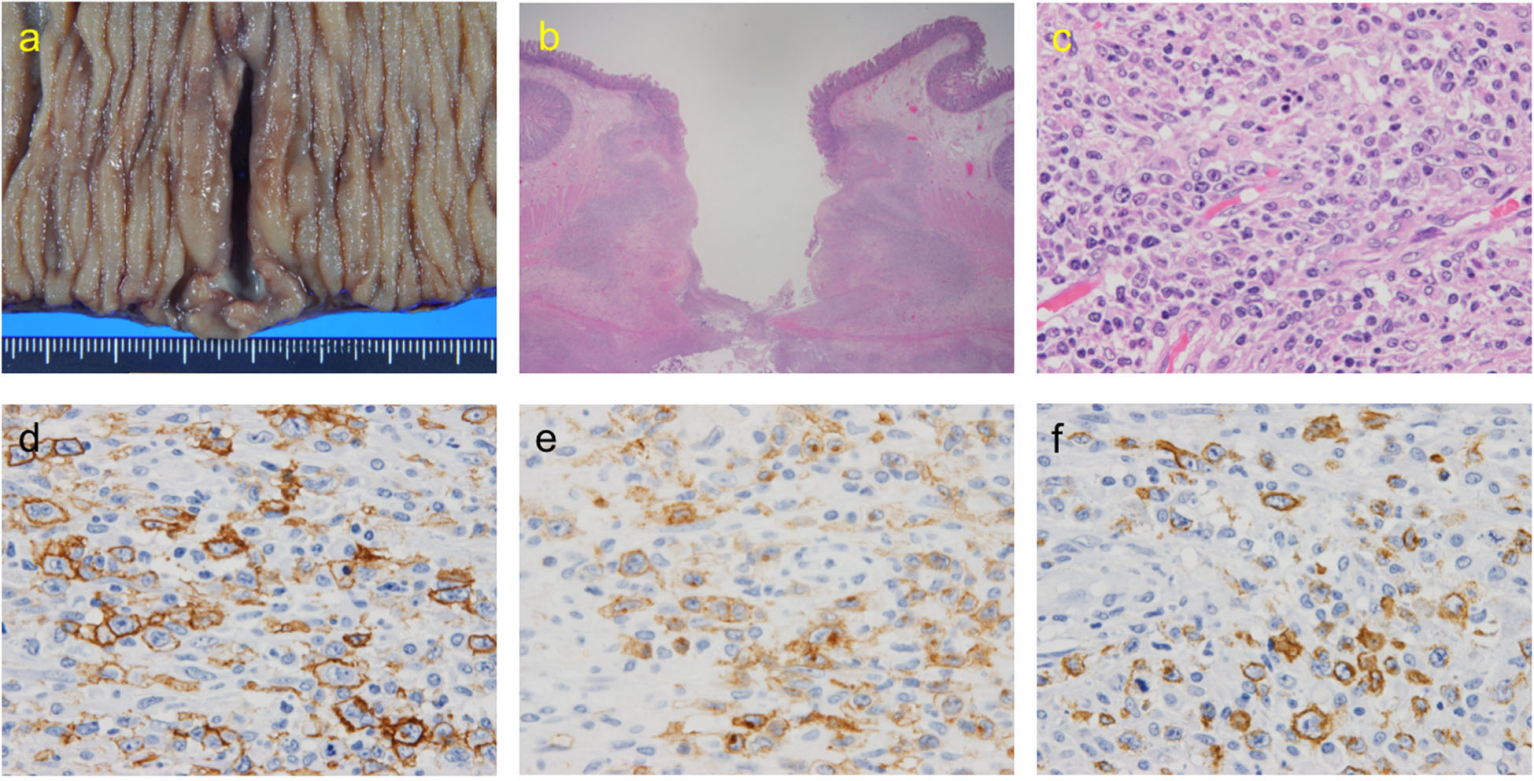
**a** Macroscopically, a 35 mm × 4 mm ulcer with a 1-mm perforation site in the jejunum. **b** Surgical specimens with perforated lesions from the ulcerated jejunum (hematoxylin and eosin staining, original magnification × 12.5). **c** Infiltration of large atypical lymphocytes around the perforated region (hematoxylin and eosin × 600). **d** Immunohistochemical staining of CD20. **e** Immunohistochemical staining of CD30. **f** Immunohistochemical staining of LMP-1 (**c**–**f** original magnification × 600). CD cluster of differentiation, LMP-1 latent membrane protein 1

The pathology of the jejunum showed a 35 mm × 4 mm ulcer and a 1-mm pinhole-shaped perforation site; infiltration of large atypical lymphocytes with small lymphocytes was observed around the perforated region (Fig. 3a–c). The immunohistochemical examination showed the expression of clusters of differentiation (CD) CD20, CD30, CD79a, and latent membrane protein 1 (LMP1) (Fig. 3d–f) and the absence of CD3, CD5, CD10, cyclinD1, cytokeratin AE1/AE3, or epithelial membrane antigen. LMP1 is a membrane protein produced by the Epstein–Barr virus (EBV), which is expressed in multiple EBV-related malignancies, including lymphomas [6, 7]. Therefore, these atypical lymphocytes were associated with diffuse large B cell lymphoma (DLBCL), a polymorphous type with Epstein–Barr virus-positive mucocutaneous ulcer (EBVMCU).

Due to the patient’s pathological findings and history of RA treated with MTX, she was diagnosed as having Oii-LPD, specifically MTX-LPD, with a histological diagnosis of EBVMCU. MTX was no longer prescribed for the patient, but oral medication was resumed on day 12, which included prednisolone (5 mg/2.5 mg every other day). The patient was discharged from the hospital on day 21, and positron emission tomography–computed tomography (PET–CT) was performed to evaluate the extent of the lymphoma, and it showed no findings associated with extranodal LPD.

**Fig. 4:**
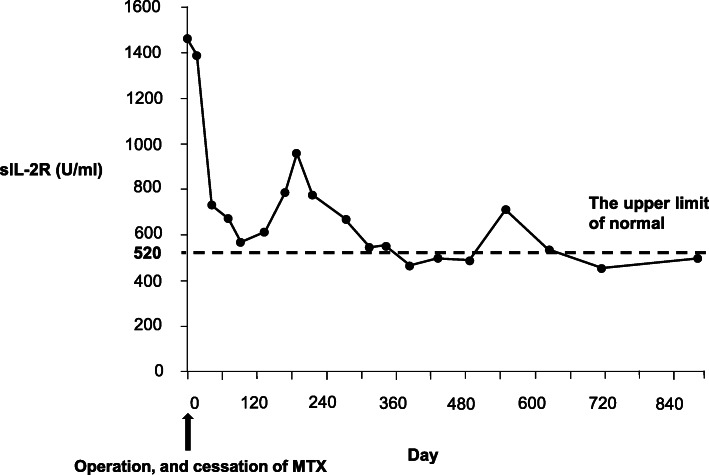
A flow chart of the clinical course for methotrexate-associated lymphoproliferative disorders

One month after the patient had concluded her MTX treatment, her soluble interleukin-2 receptor (sIL-2R) levels had decreased from 1460 to 730 IU/mL, and it was not necessary to initiate chemotherapy. One year later, her sIL-2R level was < 520 IU/mL. The patient has continued to have a good course for 2 years after her hospitalization: she has remained asymptomatic, and her sIL-2R levels show no recurrence of MTX-LPD (Fig. 4). Her RA is controlled by bucillamine (200 mg per day) and iguratimod (25 mg per day).

## Discussion and conclusions

The causes for the perforation of the small intestine can be immune-mediated, infectious- or medication-related, congenital, metabolic, vascular, or neoplastic. Medication-related causes include non-steroidal anti-inflammatory drugs; enteric-coated potassium chloride; chemotherapeutic agents, such as gefitinib and erlotinib and drug combinations containing etoposide and cisplatin; and monoclonal antibodies, such as bevacizumab, IL-2, ipilimumab, and rituximab [8, 9]. Monoclonal antibody therapeutics have been approved for several cancer and inflammatory diseases. Bevacizumab has been used in the treatment of metastatic colorectal cancer, metastatic non-small cell lung cancer, and other malignancies, including ovarian cancer, through the inhibition of vascular endothelial growth factor [9]. IL-2 has been used in patients with metastatic melanoma and renal cell carcinoma [8]. Ipilimumab is an antibody to cytotoxic T lymphocyte-associated antigen 4 that has been used in the treatment of metastatic melanoma and renal cell cancer [9]. Rituximab, an anti-CD20 monoclonal antibody, has been used to treat hematologic B cell malignancies, nephrotic syndrome, and rheumatoid arthritis [9, 10]. Several side effects associated with the use of these monoclonal antibodies have been reported, with gastrointestinal perforations reported as the rare and common adverse effects [9–12] (Table 1). Malignant neoplasms (lymphomas, enteropathy-associated T cell lymphomas, adenocarcinomas, and carcinoid tumors) can also be a causative factor of small-intestine perforation [8]. One study showed that 92 of 1062 (9%) patients with lymphoma involving the gastrointestinal tract developed a perforation [13], with large B cell lymphomas being the most common type.

**Table 1:** Indications and side effects of using monoclonal antibodies

Monoclonal antibody	Bevacizumab	Interleukin-2	Ipilimumab	Rituximab
Target	Vascular endothelial growth factor	Interleukin-2 receptor	Cytotoxic T lymphocyte antigen 4	CD20 antigen
Indications	Colorectal cancer, non-small cell lung cancer, and ovarian cancer	Melanoma and renal cell cancer	Melanoma and renal cell cancer	Hematologic B cell malignancies, nephrotic syndrome, and rheumatoid arthritis
Side effects	Hypertension, proteinuria, arterial thromboembolic events, wound healing complications, bleeding diathesis, and gastrointestinal perforations	Fever and chills, diarrhea, diffuse erythroderma, hyperbilirubinemia, anemia, thrombocytopenia, eosinophilia, a capillary leak syndrome, and intestinal perforation	Dermatitis, endocrinopathies, particularly hypophysitis, uveitis, nephritis, inflammatory myopathies, hepatitis, colitis, and intestinal perforation	Fever and chills, mucocutaneous reactions, fatal infusion reactions, progressive multifocal leukoencephalopathy, and intestinal perforation

*CD20* cluster of differentiation 20

Patients who have a therapy regimen that includes MTX can develop MTX-LPD, which can present as a benign lymphoid proliferation or malignant lymphoma. MTX-LPD is categorized as Oii-LPD by the WHO; this is a group of disorders defined as lymphoid proliferations or lymphomas that develop in patients receiving immunosuppressive drugs for autoimmune diseases or conditions other than in the post-transplant setting [14]. Extranodal lesions have been found in the digestive tracts of 4.1% of affected patients, and 1.4% had small intestinal lesions [15]. Small-intestine perforations due to MTX-LPD can occur but are extremely rare, with 7 cases reported in the Japanese literature and a single case reported in the English literature [4, 16] (Table 2). All cases were diagnosed as DLBCL, and association with EBV was observed in 5 cases. Spontaneous regression was achieved in 6 cases, and 7 cases have been reported to survive. However, a survival of > 2 years was not reported in all cases. Considering the effect of MTX discontinuation on RA progression, we should follow up cautiously.

**Table 2:** Reported cases in Japan of perforation in the gastrointestinal tract caused by MTX-associated lymphoproliferative disorder

Case	Year	Age	Sex	Organ	CD20	EBV	Diagnosis	Operation	Chemotherapy	Prognosis
1	1995	73	M	Ileum	Positive	Negative	DLBCL	Rt. hemicolectomy	No	Dead
2	2004	87	F	Ileum	Positive	−	DLBCL	Partial resection	No	Alive
3	2006	63	M	Ileum	Positive	Positive	DLBCL	Ileo-cecum resection	No	Alive
4	2011	82	F	Ileum	Positive	Positive	DLBCL	Ileo-cecum resection	Yes^a^	Alive
5	2016	70	F	Ileum	Positive	Positive	DLBCL	Rt. hemicolectomy	No	Alive
6	2017	77	F	Ileum	Positive	Negative	DLBCL	Partial resection	No	Alive
7	2018	66	F	Ileum	Positive	Positive	DLBCL	Partial resection	No	Alive
8	2020	62	M	Jejunum	Positive	Positive	DLBCL	Partial resection	No	Alive
Present case	2020	81	F	Jejunum	Positive	Positive	EBVMCU	Partial resection	No	Alive

*CD20* cluster of differentiation 20, *EBV* Epstein–Barr virus, *DLBCL* diffuse large B cell lymphoma, *EBVMCU* Epstein–Barr virus-positive mucocutaneous ulcer, *Rt. hemicolectomy* right hemicolectomy

^a^R-THP-COP 5course: rituximab (R), tetrahydropyranyl adriamycin (THP), cyclophosphamide (CPA), vincristine (VCR), and prednisolone (PSL)

The occurrence of MTX-LPD correlates with the total dose and duration of MTX administration: the median interval between initiation of MTX treatment and the development of LPD was 30–54 months, and the median cumulative dose of MTX was 940–1500 mg [17, 18]. However, another study showed that the total dose and length of MTX administration were not associated with overall survival in patients with MTX-LPD [19]. In 18–45% of cases, spontaneous recovery from MTX-LPD takes place after MTX is discontinued [3, 19], often within 2–3 months [20]. If the LPD progresses after MTX is discontinued or high sIL-2R levels persist, chemotherapy should be administered [20]. Our case achieved a disease regression without chemotherapy, although her cumulative dose (3240 mg) and duration of MTX therapy (135 months) had been much greater than the median reported in previous studies [17, 18].

Large B cell lymphoma, including DLBCL, is the major histological subtype of MTX-LPD. Other frequent subtypes are reactive lymphoid hyperplasia, classic Hodgkin lymphoma, polymorphic B cell LPD, and indolent lymphoma which includes follicular lymphoma [21]. Some studies have shown that patients who are EBV-positive and non-DLBCL histological type have a high prevalence of spontaneous remission [22]. EBV-positive DLBCL is categorized as polymorphous lymphoma and large cell lymphoma subtypes, and a number of previous reports have revealed that the polymorphous lymphoma has a good prognosis than large cell lymphoma [23]. Additionally, some patients with EBV-positive LPD exhibited mucosal or cutaneous ulcers with polymorphous infiltration of small lymphocytes, immunoblasts, and atypical large lymphocytes, which are categorized as EBVMCU. EBVMCU is typically located in the oropharynx, gastrointestinal tract, and skin [24]. Recent studies have reported that EBVMCU develops as Oii-LPD induced by MTX treatment, and nearly all EBVMCU cases showed a favorable response to conservative management approaches such as the cessation of the MTX treatment for RA [25]. Although the detection rate of EBV is 5–10% in canonical LPD, the rate increases to 27% and 30–60% in patients with MTX-LPD and rheumatoid arthritis-associated LPD, respectively [18, 22]. Among RA patients with EBV-positive MTX-LPD, a lower incidence of DNA methylation in tumors and higher expression of tumor suppressor genes were observed. Although this may explain a higher probability of spontaneous LPD regression after MTX cessation in RA patients, the causal relationship between EBV and MTX-LPD is still unclear [22]. Another study reported that the tumor microenvironment with upregulation of autophagosome may support the development of EBV-related LPD [26]. Our case had disease regression after she concluded MTX and underwent surgery for perforated jejunum. Because she had a spontaneous recovery from MTX-LPD, chemotherapy was not required; we consider that EBV-positivity played a role in the patient’s spontaneous recovery.

In conclusion, we saw a rare case of jejunum perforation induced by EBV-related MTX-LPD. Since only a few similar cases have been reported, further research is necessary to evaluate the clinicopathological features. This case study shows that successful disease management and remission can be achieved by surgery and discontinuing MTX treatment; our case did not require chemotherapy. EBV-positive patients, especially those with a pathological presentation of EBVMCU, have a higher likelihood of remission, which may have influenced the successful outcome of the present case.

## Data Availability

Not applicable.

## Abbreviations

MTX: Methotrexate
RA: Rheumatoid arthritis
LPD: Lymphoproliferative disorders
MTX-LPD: Methotrexate-associated lymphoproliferative disorders
Oii-LPD: Other iatrogenic immunodeficiency-associated lymphoproliferative disorders
WHO: The World Health Organization
CT: Computed tomography
CD: Cluster of differentiation
LMP-1: Latent membrane protein 1
EBV: Epstein–Barr virus
DLBCL: Diffuse large B cell lymphoma
EBVMCU: Epstein–Barr virus-positive mucocutaneous ulcer
PET–CT: Positron emission tomography–computed tomography
sIL-2R: Soluble interleukin-2 receptor
